# Hierarchical Modeling for Rare Event Detection and Cell Subset Alignment across Flow Cytometry Samples

**DOI:** 10.1371/journal.pcbi.1003130

**Published:** 2013-07-11

**Authors:** Andrew Cron, Cécile Gouttefangeas, Jacob Frelinger, Lin Lin, Satwinder K. Singh, Cedrik M. Britten, Marij J. P. Welters, Sjoerd H. van der Burg, Mike West, Cliburn Chan

**Affiliations:** 1 Department of Statistical Science, Duke University, Durham, North Carolina, United States of America; 2 Interfaculty Institute for Cell Biology, Department of Immunology, Eberhard Karls University, Tuebingen, Germany; 3 Program in Computational Biology and Bioinformatics, Duke University, Durham, North Carolina, United States of America; 4 Population Sciences, Fred Hutchinson Cancer Research Center, Seattle, Washington, United States of America; 5 Department of Clinical Oncology, Leiden University Medical Center, Leiden, The Netherlands; 6 Translational Oncology at the University Medical Center of the Johannes Gutenberg-University Mainz gGmbH, Mainz, Germany; 7 Department of Biostatistics and Bioinformatics, Duke University Medical Center, Durham, North Carolina, United States of America; Memorial Sloan-Kettering Cancer Center, United States of America

## Abstract

Flow cytometry is the prototypical assay for multi-parameter single cell analysis, and is essential in vaccine and biomarker research for the enumeration of antigen-specific lymphocytes that are often found in extremely low frequencies (0.1% or less). Standard analysis of flow cytometry data relies on visual identification of cell subsets by experts, a process that is subjective and often difficult to reproduce. An alternative and more objective approach is the use of statistical models to identify cell subsets of interest in an automated fashion. Two specific challenges for automated analysis are to detect extremely low frequency event subsets without biasing the estimate by pre-processing enrichment, and the ability to align cell subsets across multiple data samples for comparative analysis. In this manuscript, we develop hierarchical modeling extensions to the Dirichlet Process Gaussian Mixture Model (DPGMM) approach we have previously described for cell subset identification, and show that the hierarchical DPGMM (HDPGMM) naturally generates an aligned data model that captures both commonalities and variations across multiple samples. HDPGMM also increases the sensitivity to extremely low frequency events by sharing information across multiple samples analyzed simultaneously. We validate the accuracy and reproducibility of HDPGMM estimates of antigen-specific T cells on clinically relevant *reference* peripheral blood mononuclear cell (PBMC) samples with known frequencies of antigen-specific T cells. These cell samples take advantage of retrovirally TCR-transduced T cells spiked into autologous PBMC samples to give a defined number of antigen-specific T cells detectable by HLA-peptide multimer binding. We provide open source software that can take advantage of both multiple processors and GPU-acceleration to perform the numerically-demanding computations. We show that hierarchical modeling is a useful probabilistic approach that can provide a consistent labeling of cell subsets and increase the sensitivity of rare event detection in the context of quantifying antigen-specific immune responses.

## Author Summary

The use of flow cytometry to count antigen-specific T cells is essential for vaccine development, monitoring of immune-based therapies and immune biomarker discovery. Analysis of such data is challenging because antigen-specific cells are often present in frequencies of less than 1 in 1,000 peripheral blood mononuclear cells (PBMC). Standard analysis of flow cytometry data relies on visual identification of cell subsets by experts, a process that is subjective and often difficult to reproduce. Consequently, there is intense interest in automated approaches for cell subset identification. One popular class of such automated approaches is the use of statistical mixture models. We propose a *hierarchical* extension of statistical mixture models that has two advantages over standard mixture models. First, it increases the ability to detect extremely rare event clusters that are present in multiple samples. Second, it enables direct comparison of cell subsets by aligning clusters across multiple samples in a natural way arising from the hierarchical formulation. We demonstrate the algorithm on clinically relevant *reference* PBMC samples with known frequencies of CD8 T cells engineered to express T cell receptors specific for the cancer-testis antigen (NY-ESO-1) and compare its performance with other popular automated analysis approaches.

## Introduction

### Model-based analysis for cell subset identification in flow cytometry

Flow cytometry is the prototypical assay for multi-parameter single cell analysis, and is essential in vaccine development, monitoring of T cell-based immune therapies and the search for immune biomarkers. In many clinical research applications, the cell subsets of interest are *antigen specific* T lymphocytes that are often found in extremely low frequencies (0.1% or less). These antigen-specific T cells can be detected using HLA-peptide multimers or by their expression of effector proteins upon specific antigen stimulation in intracellular staining (ICS) assays. Current methods of flow cytometry analysis rely on visual gating of cell events to identify and quantify cell subsets of interest. However, the choice of sequence for the dot plots (gating strategy) and where to draw the gating boundaries is highly dependent on assay protocols and operator experience and may not be easily harmonized, as illustrated in recent international proficiency panels [1], [2].

There has therefore been increasing interest in the use of objective, automated methods for cell subset identification [3]. One approach that we and others have promoted is the use of statistical models to estimate the data distribution [4]–[6], followed by a mapping of summaries of the statistical distribution to cell subsets of biological interest. This model-based approach tends to be more numerically intensive than other *ad hoc* approaches to data clustering, but as we have previously demonstrated, this can be overcome by exploiting the cheap massively parallel capabilities of modern graphical processing units (GPUs). Importantly, the model-based approach has the advantage of using a declarative probabilistic framework that can be extended using well-established and understood mechanisms to improve discriminative power. In particular, hierarchical models that incorporate information from both the individual and group levels when fitted to flow cytometry data samples can increase both interpretability and sensitivity. These hierarchical models increase interpretability by aligning clusters in a way that enables direct comparison of cell subsets across data samples, and increase sensitivity for detecting very low frequency cell subsets by sharing information across multiple samples. Hierarchical models thus improve the ability of model-based approaches to detect low frequency event subsets, and enable the comparative analysis that is essential to any downstream analysis of multiple data samples.

We briefly describe three alternative software packages for automated analysis to contrast the approach of HDPGMM. FLOCK 2.0 (FLOw cytometry Clustering without K) [7] is widely used because it is a resource provided by IMMPORT (Immunology Database and Analysis Portal), a repository of data generated by investigators funded through the NIAID/DAIT. Similar to DPGMM and HDPGMM, FLOCK is able to estimate the optimal number of data partitions from the data. However, FLOCK uses an adaptive multi-dimensional mesh to estimate local density followed by hierarchical merging of adjacent regions based on density differentials rather than a mixture model, and does not appear to either provide a statistical model (e.g. for goodness-of-fit calculations) or methods for alignment of cell subsets across different samples. In contrast, flowClust [6] and FLAME (FLow analysis with Automated Multivariate Estimation) [5] both use a statistical mixture model approach for density estimation and clustering. Both packages are likely to be widely used, since flowClust is provided as a library in R/BioConductor, and FLAME is part of GenePattern. Apart from the choice of base distribution (T distribution for flowClust and skewed distributions for FLAME), the main differences with DPGMM are the use of optimization (Expectation-Maximization) rather than simulation (MCMC) to estimate the density, the need for the user to specify the number of partitions and differences in the type of transform applied in data pre-processing. FlowClust does not provide any method to align cell subsets across samples, while FLAME provides a heuristic algorithm to do so as described in their original publication [5]. Unlike HDPGMM, none of the three algorithms use a hierarchical approach to model group and individual specific effects.

With this in mind, the developments reported here concern the implementation of a hierarchical Gaussian mixture model based on a Dirichlet process prior, and extensions of the basic model to identify and quantify rare cell subsets in flow cytometry data. Simulated data is first used to demonstrate the advantages of hierarchical models over conventional clustering approaches. This is followed by validation of the model on experimental samples, using retrovirally TCR-transduced T cells that are spiked into autologous peripheral blood mononuclear cell (PBMC) samples to give a defined number of antigen-specific T cells [8]. Finally, the reproducibility and accuracy of this approach for rare cell quantification is compared to that of standard DPGMM and manual analysis performed by a group of ten flow cytometry users, and compared with the results from FLOCK, FLAME and flowClust.

### Statistical mixture models

The basic concept in model-based approaches is to consider events in a flow cytometry data set as being random samples drawn from a multi-dimensional probability distribution. The objective of analysis is then to define the probability distribution model and evaluate inferences over the model parameters based on fit to the specific data set. Statistical mixture models are a standard approach for the construction of the underlying distribution, using the sum of many simpler probability distributions (e.g. multivariate Gaussian, Student-t or skewed distributions) to approximate arbitrary multi-dimensional distributions. For biological interpretation, fitted models are then used for clustering, i.e. using statistical properties of individual events to assign them to biological cell subsets. For example, with statistical mixture models, this can be done by grouping events with the highest probability of coming from a specific mixture component together, or merging of multiple components using specified criteria such as having a common mode in the estimated distribution over markers [9], [10].

Of course, the number of distinguishable cell subsets and Gaussian components necessary to fit the model satisfactorily is not known in advance. To avoid having to specify the number of mixture components needed in the model, we use a Dirichlet process prior in which the number of components necessary is directly estimated from the data [11]. Computationally, the use of Dirichlet process priors is more efficient than fitting multiple models with different numbers of components and testing with some penalized likelihood (e.g. Akaike or Bayesian information criteria) to choose the best model, as only a single model fit is performed. Since we use multivariate Gaussian distributions as components, the overall approach is described as a Dirichlet process Gaussian mixture model (DPGMM). DPGMM are extremely flexible models that can fit flow data from flow cytometry experiments using different antibody-fluorochrome labels (e.g. 4-color HLA-peptide multimer and 11-color intracellular staining (ICS) panels), and a natural evolution of the fixed 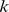 Gaussian mixture models we originally proposed [4]. Finally, while the model uses Gaussian components, cell subsets are identified with *merged* components using the consensus modal clustering strategy described in Methods. As a result, cell subsets can have arbitrarily complex distributions and are not restricted to symmetric Gaussian clusters.

### Limitations of clustering approaches

Clustering methods applied to data samples independently face two major limitations. The first is that cluster labels are not aligned across data samples, posing a problem for comparing subsets across multiple samples which is usually the purpose of the original experiment. The second is that there are limits to the ability of clustering models to identify very rare event clusters due to *masking* by abundant event clusters [12]. In particular, this makes it difficult to identify clusters matching antigen-specific HLA-peptide multimer labeled or polyfunctional T cells in ICS assays that may be biologically meaningful at frequencies of 0.1% or lower. We show in this paper that both issues are successfully addressed by the use of hierarchical Dirichlet process Gaussian mixture models (HDPGMM).

### Hierarchical Dirichlet process for information sharing

Hierarchical, or multi-level models, represent individual events in flow cytometry data as being organized into successively higher units. For example, individual events belong to a sample, and a sample may belong to a collection of similar samples. The critical idea is that cell subset phenotypes that are common across data samples can be used to inform and hence better characterize events in individual samples. For example, one hierarchical Dirichlet process model formulation partitions components into those common across data samples and those unique to a specific sample [13], [14] – this provides a different notion of sharing that is useful for identifying fixed and variable components across heterogeneous data samples but lacks a straightforward alignment of all clusters necessary for multi-sample comparison.

Instead, we model information sharing by placing all data samples under a common prior, such that the mean and covariance in any of the individual sample Gaussian components are shared across all samples, but the weight (proportion) of the component in each sample is unique. As described by Teh et al (2006) [15], this can be achieved by using a set of random measures 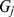, one for each data sample, where 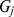 is distributed according to a sample-specific Dirichlet process 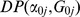. The sample-specific DPs are then linked by a common discrete prior defined by another 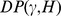. This hierarchical model leaves the cluster locations and shapes constant across datasets, and hence aligns the clusters in that the location of the normal components is common to all data samples.

**Figure 1:**
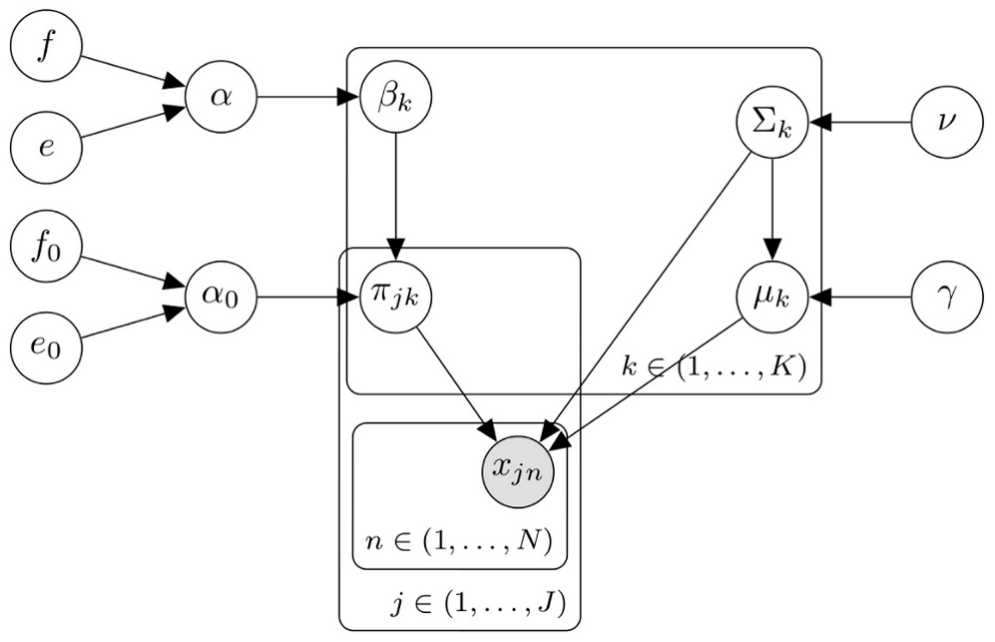
Schematic summary illustrating the HDP model framework. A graphical model provides a declarative representation of the HDPGMM. The figure shows a compact plate representation of the graphical model, in which plates (rounded rectangles) are used to group variables in a subgraph. Each subgraph in a plate is replicated a number of times as indicated by the label within the plate. The 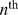 event in the 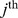 sample is represented by 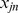, and the 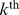 component for the 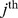 sample is a multivariate Gaussian with proportion 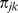, mean 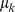 and covariance matrix 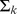.s. Hyper-parameters that can be set are 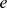, 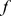, 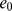, 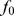, 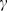 and 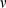 as described in Methods. Given the declarative graphical model, standard and GPU-accelerated MCMC sampling algorithms can be used to implement the model as previously described [16].

As depicted in the summary schematic of the HDPGMM model shown in Figure 1, there are basically 6 parameters that control the sensitivity. The parameter 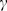 controls the spread of the (standardized) cluster means and 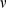 controls how informative our prior is about the shape of the covariances. The default for these parameters is vague and it is our opinion that 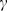 and 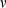 should not be tuned since it is unlikely that a user is knowledgeable about these constraints. The next set of parameters 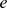 and 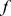 are hyper-parameters for the Gamma distribution on 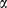 which controls the overall number of clusters. Small values of 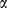 will encourage fewer clusters and large values of 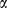 will encourage more clusters. The mean and variance of the Gamma distribution are 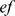 and 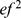 respectively, and the default is set such that both mean and variance are 1. As an example of how we can tune this, if we set 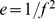, the variance will be fixed, and the mean will vary as 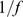 – in that case we can encourage larger values of 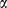 and more clusters by choosing small values of 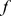. The final set of parameters 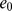 and 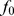 are hyper-parameters for the Gamma distribution on 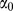 which specifies how similar the weights for each sample are to the other samples' distribution – when 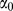 is small, the amount of information shared is small (weights for each batch can be very different from the overall distribution); when 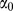 is large, the weights for each batch are likely to be similar to the base distribution. Tuning of 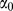 via 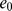 and 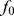 is analogous to tuning 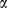 via 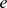 and 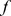.

In the context of flow cytometry, a data sample typically consists of an 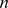 by 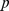 data matrix from a single FCS file, where there are 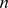 events and 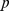 features reporting scatter and fluorescent intensities. The HDPGMM is a model that fits a collection of such data samples, and makes the assumption that the same cell subsets are present in every sample with frequencies that vary from sample to sample. The model does not make any further assumptions about whether the samples in a collection come from the same or different subjects, experimental conditions, treatment groups etc. Different flow cytometry technologies generate data sets that mainly vary in the maximum number of features that can be observed rather than in the standardized locations of cell subsets or their covariances, and hence 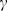 and 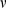 do not need tuning. With more features, it is likely that more cell subsets can be distinguished, and it would be reasonable to tune 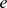 and 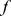 to encourage larger values of 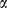. The values of 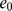 and 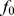 do not depend on the flow cytometry technology, but rather on how similar or different samples are from each other, and can be tuned accordingly. The number of mixture components that are needed for a good model fit is also likely to increase, and we present a diagnostic for model goodness-of-fit that can be used to guide choice of the lower bound for the number of components used in the results and discussion.

The hierarchical DP mixture model allows information sharing over data sets. In the hierarchical model, each flow cytometry data sample can be thought of as a representative of the collection of data samples being simultaneously analyzed. The individual data samples then provide information on the properties of the collection, and this information, in turn, provides information on any particular data sample. In this way, an HDPGMM fitted to a single data sample “borrows strength” from all other samples in the collection being analyzed. In other words, if a rare cell subtype is found in more than one of the samples, we share this information across the samples in the collection to detect the subtype even though the frequency in a particular data sample may be vanishingly small. HDPGMM thus increases sensitivity for clustering cell subsets that are of extremely low frequency in one sample but common to many samples or present in high frequency in one or more samples. In principle, there is no lower limit to the size of a cluster that can be detected in a particular sample. In practice, vanishingly small clusters (e.g. 3–5 events out of 100,000) require expert interpretation to distinguish background from signal, but it is not uncommon for biologically significant antigen-specific cells to be present at such frequencies.

## Results

### Simulation study

**Figure 2:**
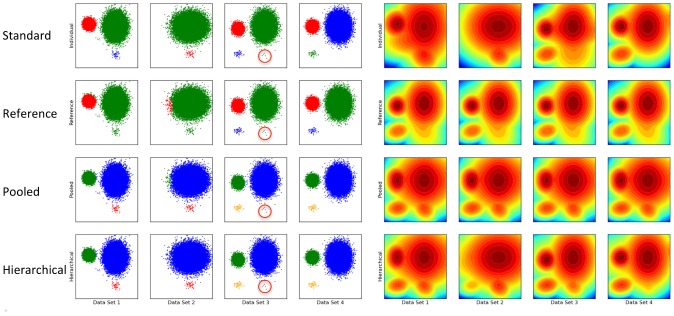
HDPGMM results in more accurate classification of events in simulated data than other statistical mixture model approaches. (Left) Row 1 shows independent fitting of DPGMMs to each data set; row 2 shows the use of reference posterior distribution from data set 3 to classify events in other data set; row 3 shows a DPGMM fitted to pooled data from all data sets; and row 4 shows fitting of an HDPGMM to all 4 data sets. Results are described in the text. Within each row, if two events are assigned to the same cluster, they are given the same color - it can be seen that clusters are aligned in Rows 2–4, but not in Row 1. All models used a truncated DPGMM base with 16 components, a burn-in of 10,000 iterations, and sampling of 100 post burn-in iterations for the calculation of the posterior distribution. (Right) Contour plots of the log posterior distribution. The HDPGMM distributions (Row 4) are most similar to the independently fitted distributions (Row 1), with the advantage that the small cluster in data set 3 masked by its larger neighboring cluster on top has a distinct mode. In contrast, the reference and pooled distribution strategies have the exact same distribution for all data sets and lack the flexibility to model sample-specific features.

We illustrate the ability of hierarchical modeling to simultaneously overcome the problem of masking of rare event clusters and provide an alignment of cell subsets over multiple data samples. Four simulated data sets were created, each with up to 4 bivariate normal clusters in 4 quadrants. Clusters in each quadrant may have different means or covariance matrices, or be absent entirely; see Figure 2. We compared four different approaches to clustering the data – independent fitting of DPGMM to each data sample, using a reference data set, using pooled data, and using hierarchical modeling.

#### Independent fitting of DPGMM to simulated data

**Figure 3:**
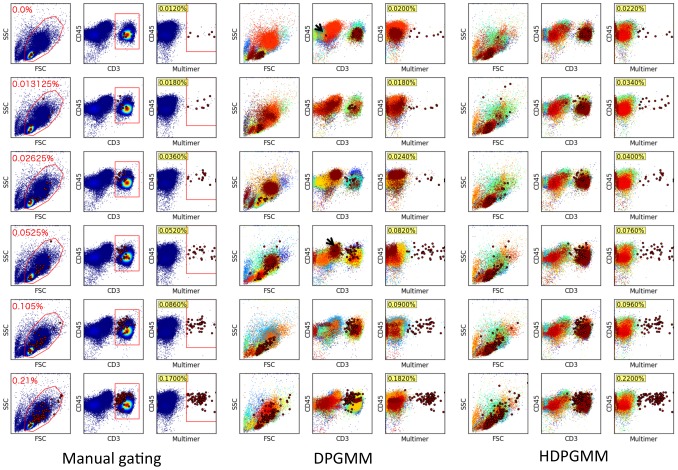
Comparison of manual, DPGMM and HDPGMM detection of rare antigen-specific events. The panels show the estimated frequencies of antigen-specific cells (large red dots) expressed as a percentage of all events (yellow boxes). These percentages were estimated using manual gating by a representative user (left), DPGMM (middle) and HDPGMM (right). Text in red in the first column shows the spiked-in frequency of retrovirally transduced T cells for the data sample in that row. The red polygons in the left panel are gates used for identifying antigen-specific cells by manual gating; the exact shape, sequence and location of these gates is determined by the operator and may vary between different operators depending on their training, experience and expertise. With the DPGMM approach, cell subsets across the samples from top to bottom are not directly comparable as indicated by the event colors, posing a problem for quantification of the same cell subset in different samples. In contrast, with the HDPGMM approach, cell subsets are aligned and directly comparable across all samples. HDPGMM is more sensitive at detecting antigen-specific cells when the frequency is extremely low (first 3 rows). HDPGMM is also more consistent in labeling events across different samples, while DPGMM is prone to detect likely false positive antigen-specific cells that are CD3-low or negative (arrows in rows 1 and 4 of middle panel). HDPGMM improves on the accuracy and consistency because the model incorporates both sample-specific and group-specific information, in contrast to DPGMM which only has access to sample-specific information. For both DPGMM and HDPGMM, model fitting was done with an MCMC sampler running 20,000 burn-in and 2,000 averaged iterations.

In Figure 2 row 1, DPGMM were independently fitted to each of the data samples and modal clustering performed on the posterior distribution averaged over post burn-in iterations. Events were assigned to the modal clusters for which the posterior probability was highest and color coded by the identity of the modal cluster. The first obvious issue with this approach is that modal cluster labels are not coherent over data samples, as shown in the top row of Figure 2, and also by the different assignments of similar cell subsets in different samples in the middle panel of Figure 3. Consequently, it is not possible to directly compare modal cluster frequencies across data sets without further post-processing. The second more subtle issue is that the small (5 event) cluster in data sample 3 (circled in red) has been masked by the large green cluster even though it matches the distinct blue cluster in data sample 1 and the red cluster in data sample 2 and should be interpreted as a separate cell subset.

#### Using a reference data set

In row 2, we fitted a DPGMM to sample 3 (reference data set), then used the posterior distribution found to classify events in all the other samples. While this ensures that all clusters are aligned across the data sets, it has several limitations. The first issue is the need to choose a specific reference data sample, which introduces an element of subjectivity. A more worrying issue is that differences in distribution across data samples are simply ignored, and this can result in artifacts as shown with data sample 1 and data sample 2, where there is mixing of the red/green clusters because the mean or covariance matrices of those clusters deviated from that of the reference data sample 3. Also, because the small cluster (circled in red) is masked in data sample 3, it is also missed in all the other samples. While another data sample could have been chosen as the reference, it is clear from inspection of the variation across the simulated data samples that no single reference can give a satisfactory result.

#### Using pooled data

In row 3, we fitted a DPGMM to pooled data from all four data samples. Pooled data is problematic because the resulting distribution is for an “averaged” data sample, and may result in the loss of information specific to a particular sample. We observe artifacts from clusters present in the pooled distribution but not in the specific sample in data sample 2 (green events in blue cluster) and data sample 4 (red events in blue cluster). A subtle issue is the over-counting of red cluster events in data sample 3 (9 events circled in red) due to the excessive influence of the red clusters in data samples 1 and 2.

#### Hierarchical modeling

Finally, in row 4, we fitted a HDPGMM to all four data sets simultaneously with the consensus modal clustering approach to identify cell subsets as described in Methods. Clusters are aligned across data sets, there is no spurious mixing of clusters, and the rare event cluster in data sample 3 is correctly classified as having 5 events (circled in red).

### Experimental study

To evaluate the utility of HDPGMM for identifying rare event clusters in real data, we used reference cell samples containing a predefined number of T cells with known TCR specificity for the NY-ESO-1 cancer-testis antigen. TCR-transduced cells were added to autologous PBMC samples at final concentrations of 0%, 0.013125%, 0.02625%, 0.0525%, 0.105% and 0.21% [8]. There is also a small background contribution by antigen-specific T cells that are already present in the unspiked sample, which is estimated to be 0.0154% using the mean frequency from manual gating by 10 flow practitioners. A total of 50,000 events was then collected from each sample for analysis. At the highest spike frequency, we would therefore expect to detect a maximum of 0.2254%, or 113 antigen-specific T cell events out of 50,000 total events. This is a challenging clustering problem as the frequency of expected multimer-positive events is extremely low, but ideal for validation since the expected number of T cells that bind with high-affinity to the HLA-peptide multimer is known.

**Figure 4:**
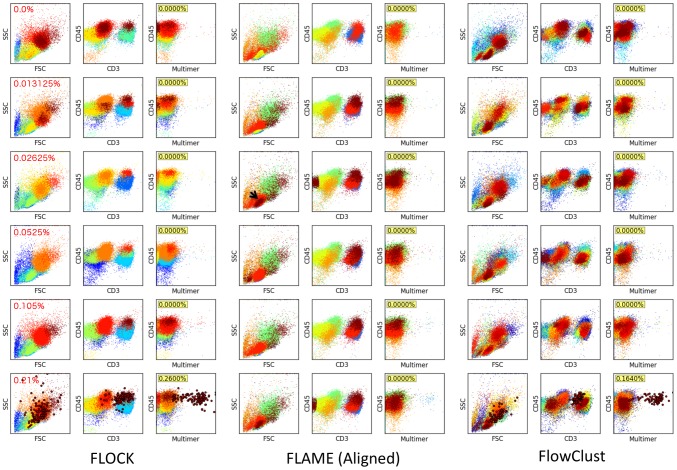
Trace plots of log likelihood for the final 2,000 MCMC iterations of the HDP model sampled every 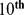 iterations showing mixing and convergence. The MCMC was run for 22,000 iterations, and samples obtained from the final 2,000 iterations were used to calculate and plot the log likelihood at each iteration. The log likelihood appears to vary stochastically about an equilibrium distribution indicating convergence, and the chain traverses its distribution indicating mixing, but the steps tend to be small indicating some degree of autocorrelation. Text in yellow boxes indicates the frequencies of the spiked antigen-specific T cells in the sample being fitted.

**Figure 5:**
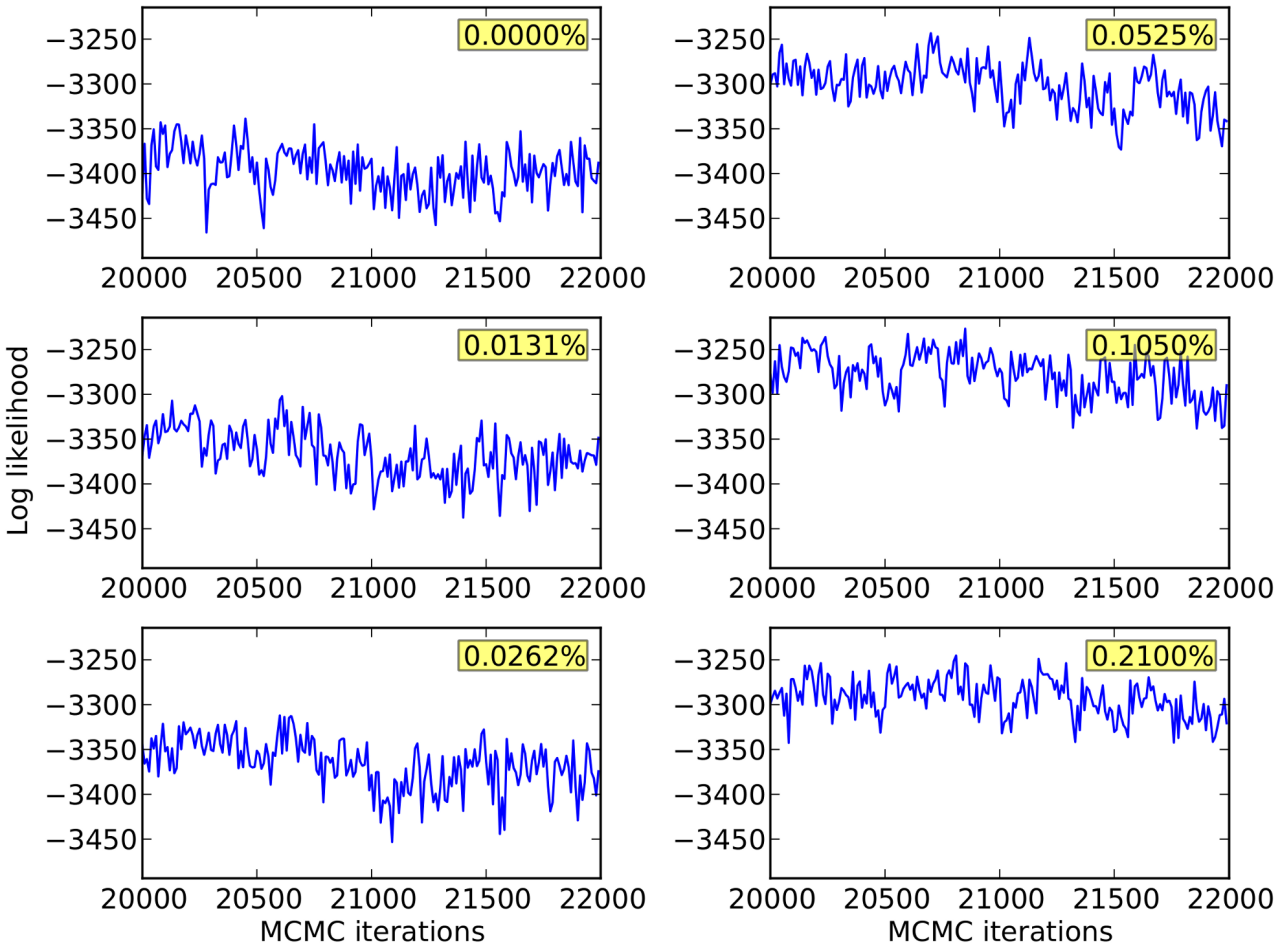
Log of the proportion or weight of each of the 128 components in the model. Each panel shows a scatter plot of the log component proportions ordered by size for the HDP model fitted to each flow cytometry data sample. The largest component has a log probability of approximately -1, indicating that this single component can account for about 10% of the total events in the data sample. In contrast, the smallest component has a log probability of between -5 and -6, indicating that the smallest component only accounts for 0.001–0.0001% of the total events in the data sample. Since each sample has 50,000 events, components with log probabilities of -5 and below are likely to be empty of events. Hence, the dip at the right of each plot is an indication of cutting back by the Dirichlet process model, and provides evidence that the number of components is adequate for a good model fit. If there is no dip in the size of smallest component proportions, there is a need to increase the maximal number of components if rare event clusters are to be adequately modeled. Text in yellow boxes indicates the frequencies of the spiked antigen-specific T cells in the sample being fitted.

DPGMM and HDPGMM models were separately fitted to these six data samples using the FSC, SSC, CD45, CD3 and HLA-multimer channels (5 dimensional), using a truncated Dirichlet process with 128 mixture components, 20,000 burn-in steps and 2,000 identified iterations to calculate the posterior distribution as described in Methods. The trace plots of log-likelihood shown in Figure 4 provides evidence for model convergence, and the distribution of mixture component proportions in Figure 5 provides evidence for model goodness of fit. After consensus modal clustering, the multimer positive clusters were defined using the gating scheme shown in the left panel of Figure 3, but applied to event clusters found by HDPGMM rather than individual events. Since the clustering is done in the full set of markers rather than in two-dimensional slices, events that look close together in a particular projection but are further apart when all dimensions are considered will not belong to the same cluster. The frequency of multimer-positive events as a percentage of all 50,000 events was then calculated. We also ran trials of HDPGMM to evaluate the lower bound needed to find the antigen specific clusters in all samples; 3 out of 4 runs were successful with 32 components, and all runs were successful when 40 or more components were used.

A side-by-side comparison of manually gated, DPGMM and HDPGMM classifications is shown in Figure 3. All 3 approaches are comparable in terms of being able to identify and quantify the antigen-specific cluster of events. Across all runs, DPGMM consistently finds occasional outlier events that are likely to be false positives (e.g. the CD3 negative to low events in the DPGMM fits shown in rows 1 and 4). HDPGMM does not appear to suffer from the same false positive detection, and is also more sensitive for the samples with the lower spiked-in frequencies than DPGMM. However, the most striking advantage of HDPGMM over DPGMM is the interpretability of the hierarchical modeling – cell subsets are consistently labeled across data samples, allowing direct comparison of any cell subset of interest, not just of the multimer positive events.

**Figure 6:**
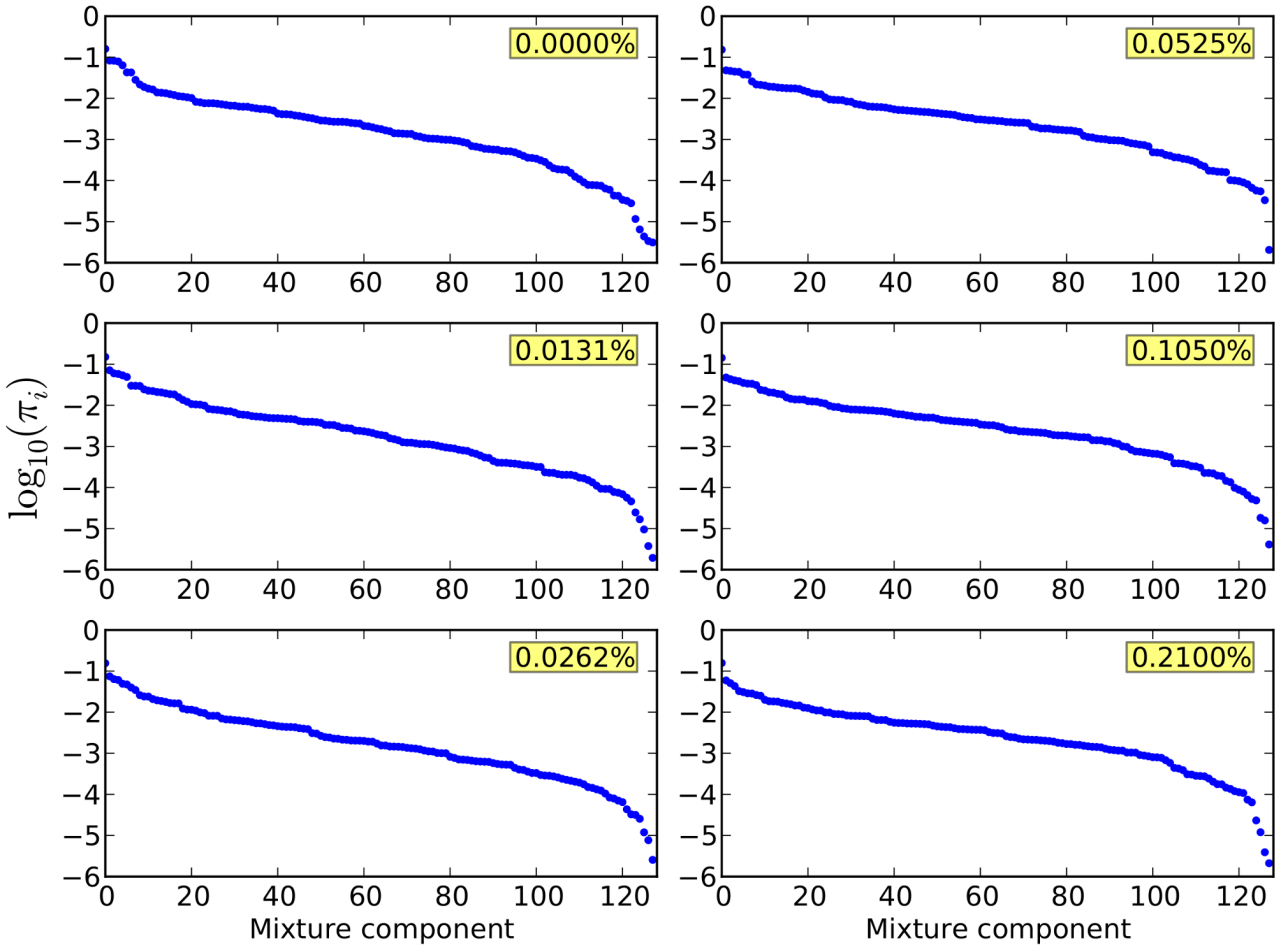
Comparison of FLOCK, FLAME and flowClust for detection of rare antigen-specific events. The panels show the estimated frequencies of antigen-specific cells (large red dots) expressed as a percentage of all events (yellow boxes). (Left panel) FLOCK detects the antigen-specific cluster at the highest spiked-in frequency but not in the other samples. There are several CD3-negative events included in the detected cluster that are most likely false positive events. As indicated by the color coding of events, FLOCK does not provide any alignment of cell subsets across samples. (Middle panel) Using the default settings, FLAME failed to identify any antigen-specific cell subsets. Cell subsets found were aligned but there were alignment artifacts when the event partitioning was different across samples (arrowed example). (Right panel) Using 64 components and 1000 iterations, flowClust only identified antigen-specific clusters at the highest spiked-in levels and did not provide any methods to align clusters across samples.

Figure 6 shows the results from the application of FLOCK, FLAME and flowClust on the same data set. FLOCK only detects the antigen-specific cell subset at the highest spiked-in concentration with a moderate number of probable false positive events that are CD3-negative. As indicated by the color coding of events, FLOCK does not provide any alignment of cell subsets across samples. Using the default settings, FLAME failed to identify any antigen-specific cell subsets. Cell subsets found were aligned but there were alignment artifacts when the event partitioning was different across samples (arrowed example). Using a 64 component mixture, flowClust only detects antigen-specific clusters at the highest spiked-in concentration, and does not provide any alignment of cell subsets. Unlike FLOCK and Dirichlet process based models, the number of components for FLAME and flowClust is not estimated from the data. Hence, in practice, one would have to fit a variety of models with different numbers of components and subsequently perform model selection when using FLAME or flowClust.

In Figure S1, we compare HDPGMM, FLAME and flowClust models with 48 components fitted to the same data set. HDPGMM completed in 3 hours and 30 minutes (20,000 burn-in and 2,000 identified iterations), FLAME took 4 days 12 hours and 28 min, and flowClust completed in 25 minutes (1,000 iterations). With 48 components, HDPGMM found antigen-specific clusters in all samples. FLAME found the clusters when the spiked in concentration was greater or equal to 0.02625%, but cluster alignment failed with the error “missing value where TRUE/FALSE needed”. In contrast, flowClust did not detect any antigen-specific clusters. Both HDPGMM and FLAME clusters included a fair number of CD3-negative events, in agreement with the goodness-of-fit analysis shown in Figure 5 that 48 components is inadequate for modeling rare event clusters in this data set. We tried to run FLAME with 128 components but this was not practical since the program did not terminate after more than 10 days. It took 26 hours for flowClust to run 1,000 iterations with 128 components, and 4 out of 6 samples gave “NA” indicating missing data for all cluster centroids. The wide variation in run-times seen with flowClust (25 minutes to 26 hours) probably reflects early termination with fewer than 1,000 iterations due to tolerance thresholds being met in the 48 component case. We suspect that the missing data might be caused by the Expectation-Maximization algorithm failing when there are zero-event components, but cannot confirm this since the program terminated with no error messages.

**Figure 7:**
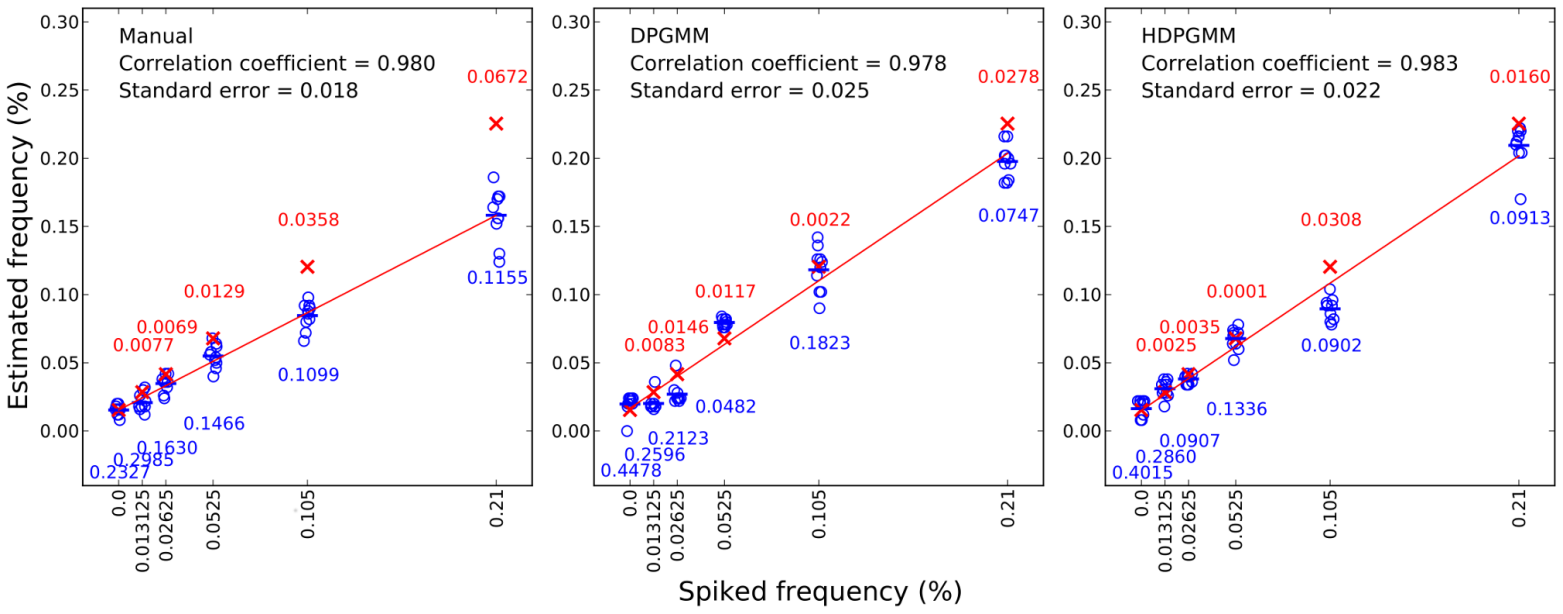
Comparison of accuracy, reproducibility and linearity of manual gating, DPGMM and HDPGMM. For gating estimates, frequency estimates from 10 flow cytometry operators were collected. For both DPGMM and HDPGMM, 10 MCMC runs with unique random number seeds were performed to evaluate the reproducibility of antigen-specific cell frequency estimates. Estimates of the antigen-specific frequencies from manual, DPGMM and HDPGMM approaches are shown as open blue circles, with the blue bar representing the mean of all 10 estimates at each spike frequency. The red crosses represent the “true” frequency of antigen-specific cells combining the known spiked-in frequencies and the average background from 10 manual evaluations. As shown in the figure, HDPGMM (right panel) estimates have equal or less variability at every spike dilution when compared with DPGMM (middle panel). A linear regression fit (red line) shows that the standard errors and correlation coefficient of all 3 approaches are comparable. The number in red text above each set of estimates is the absolute value of (median of estimates – “true value”), a measure of accuracy. This shows that HDPGMM is more accurate than manual gating at every spiked-in concentration. The number in blue text below each set of estimates is the coefficient of variation (CV), which is lower for HDPGMM than manual gating for all concentrations except autologous sample only. For both DPGMM and HDPGMM, model fitting was done with an MCMC sampler running 20,000 burn-in and 2,000 averaged iterations.

**Figure 8:**
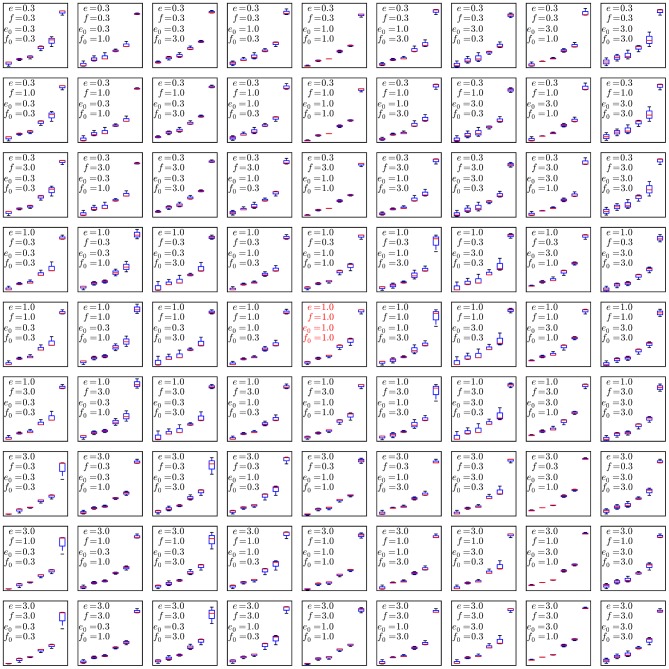
Sensitivity analysis for the 4 configurable hyper-parameters 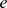
 **,** 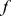 **,** 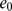 **and** 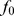 **.** To evaluate the robustness of the algorithm to changes in the configurable hyper-parameters, we repeated the analysis of the spiked in data sample multiple times with different parameters, using 10 independent MCMC runs to obtain statistics for each set of hyper-parameter configurations. Each mini-panel has the same axes as Figure 7 with estimated frequency of multimer-positive events on the vertical axis and spiked-in frequency on the horizontal axis. A boxplot is used to display the results for each model configuration. Configurable parameters were set to be either the default value (1.0), 3-fold lower (0.3) or 3-fold higher (3.0), giving 81 hyper-parameter configurations. Three replicate runs with 10,000 burn-in and 1,000 MCMC iterations were performed for each configuration. The default configuration is in the center panel with red text.

Finally, to evaluate the robustness of the DPGMM and HDPGMM frequency estimates, the fitting was repeated 10 times for each algorithm using different random number seeds, and compared to manual gating results from 10 users. Manual gating was performed by operators who were instructed to gate using the same sequence of 2D plots (common gating strategy), but were free to set gate boundaries within any given plot. The results are shown in Figure 7. With respect to linear regression, all three methods perform comparably well with respect to correlation coefficient, but manual gating has slightly less deviation from a straight line fit than HDPGMM which in turn is better than DPGMM. From the figure, it can also be seen that HDPGMM is more accurate than manual gating in that the absolute deviation of the median of the estimates from the “true” concentration is lower than that for manual gating at every concentration. Since the “true” value is taken to be background estimated from 10 manual estimates in the autologous PBMC only sample added to the known spiked-in frequency, accuracy is not evaluated for autologous sample alone. In Figure 8, we show that the algorithm is robust to changes in the hyper-parameters across a 9-fold range.

## Discussion

We have shown that HDPGMM improves on fitting individual samples with DPGMM in two ways - 1) it aligns clusters, making direct comparison of cluster counts across samples possible, and 2) by sharing information across samples, it can identify biologically relevant cell subsets present at frequencies in the 0.01–0.1% range, since “real” cell subsets would naturally be expected to be present in multiple data samples. The hierarchical model is also preferable to using a reference data sample or pooling the data from all samples, since individual sample characteristics are lost with these alternative strategies.

Unlike HDPGMM, other approaches for automated flow cytometry analysis treat data in the same way as DPGMM, that is, fitting a model to independent samples separately, then using a heuristic or algorithm to match up clusters in one data set with another. However, since the model fitting is performed independently, the way that events are partitioned in individual data sets into clusters may be different even across samples that are otherwise very similar, resulting in poor alignment as seen in the FLAME analysis. We are not aware of any other automated flow cytometry analysis software that directly models contributions from individual and grouped samples to align cell subsets, and believe that the HDPGMM approach fills a useful niche in multi-sample comparisons, especially for the quantification of rare event clusters.

One limitation of the HDPGMM model is that all the data to be fitted need to be simultaneously available. This is not an issue for most studies, but may be limiting for longitudinal studies that collect samples serially over an extended period where interim analyses need to be performed. Even in these cases, it may be useful to batch process cell samples in stages using a hierarchical model, then perform post-processing to align cell subsets over different stages. Because of information sharing, cell subsets that are consistent across data samples will be extremely robust features in the posterior distribution. Hence, it is likely that features across batches will be more consistent and easier to align for HDPGMM-fitted batch samples than if every sample was independently fitted.

As described in the text, HDPGMM achieves alignment by assuming that the cluster locations and shapes are constant across datasets, and only their proportions vary from sample to sample. This is similar to the standard practice of using a gating template common to all samples for manual analysis. However, the HDPGMM approach has several advantages over the use of a common gating template. Because the locations and shapes of the clusters are inferred from the data and not imposed top-down by an expert, there is less risk of a subjective bias and failure to detect novel cell subsets. Since classification of events is done by assignment to the maximum probability cluster, cell subsets are not demarcated by arbitrary (typically polygonal) boundaries. In addition, it is simple to tune for higher sensitivity or specificity depending on experimental context by setting the probability necessary for an event to be included in a cluster; events that fall below this threshold are considered to be indeterminate. However, clusters that are doubly rare in the sense of being found in only a small proportion of the samples, and which also constitute a tiny fraction of the total events in any given sample, risk being masked by other more common and high abundance clusters. In many cases, this limitation can be addressed by the inclusion of appropriate positive controls in the samples. Where such positive controls are not available, a post-processing step to scan for “anomalous” events that are found in extremely low probability regions of the posterior distribution at higher frequencies than predicted, may be effective for identifying these doubly rare events.

Technically, our implementation of the HDPGMM integrates several innovations necessary to make such hierarchical models a practical tool for flow cytometry analysis, including the use of a Metropolis-within-Gibbs step for sampling, an identification strategy to maintain consistent component labels across iterations that allows us to calculate the posterior distribution from multiple MCMC iterations, and a consensus modal map to merge components in such a way that non-Gaussian cell subsets are aligned across multiple data sets. To ensure scalability, we have implemented Message Passing Interface (MPI) and Compute Unified Device Architecture (CUDA) optimized code that can take advantage of multiple CPUs and GPUs from a cluster of machines to fit a single HDPGMM model to multiple data sets.

We provide software for HDPGMM fitting to flow cytometry data sets, together with pre-specified robust default parameters and hyper-parameters that make practical usage simple. In our experience, we have never needed to adjust these parameters for data sets ranging from 3-color to 11-color flow cytometry data sets. The only parameters we individually set are the number of burn-ins, the number of iterations to collect for the posterior distribution, and the maximal number of components for the truncated DP algorithm. These parameters are tuned mainly for computational efficiency since conservative defaults that would be expected to be effective in all common use cases can be given, with the trade-off being longer run times. In addition, the use of prior information to set the starting values for component means and covariances (e.g. from fits to previously collected similar data) would reduce the number of iterations necessary to achieve convergence.

**Figure 9:**
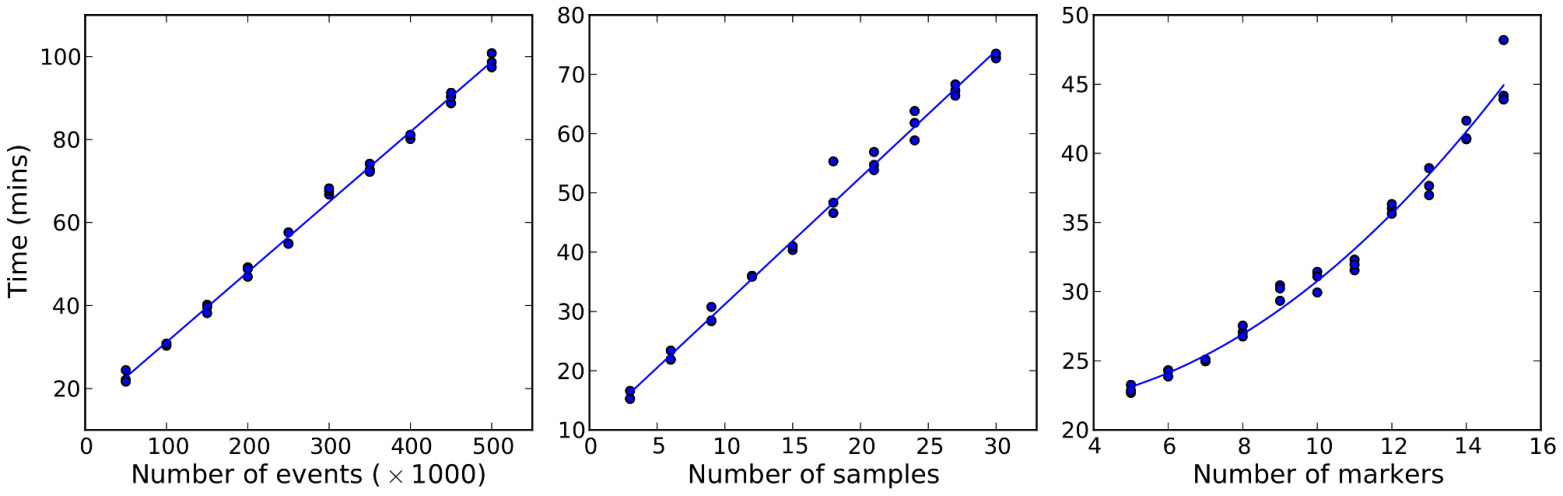
Performance of HDPGMM with different numbers of events, samples and markers. Left panel shows time taken to fit HDPGMM to 10 samples with 50,000 to 500,000 events and 10 markers. Middle panel shows time taken to fit HDPGMM to 3 to 30 samples each with 100,000 events and 10 markers. Right panel shows time taken to fit 10 samples each with 100,000 events with the number of markers varying from 5 to 15. In each case, the model was run for 1,000 MCMC steps with an upper limit of 128 mixture components on a NVidia GTX 580 GPU, and the times from three replicate runs are shown.

The fitting of HDPGMM is computationally demanding but can be accelerated with cheap commodity graphics cards as previously described [16]. For example, running an MCMC sampler for 20,000 burn-in and 2,000 identified iterations to fit a 128-component HDPGMM to the six multimer data sets shown in Figure 3 took less than 6 hours on a Linux workstation using a single NVidia GTX 580 card costing under USD 500. The algorithm has runtime complexity of 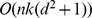, and benchmark experiments shown in Figure 9 confirm that the performance is linear in the number of events and samples and quadratic in the number of markers. Open source code for fitting DPGMM and HDPGMM models to flow cytometry data is available from http://code.google.com/p/py-fcm/. The code is written in the Python programming language, and will run on regular CPUs, but is optimized for massively parallel computing using the CUDA interface (a suitable Nvidia GPU is required for CUDA). Flow cytometry data samples, source code and a sample script to fit a HDPGMM model to the data are provided in Supplementary Materials.

In summary, we describe and provide code for a hierarchical modeling extension to statistical mixture models that improves on the robustness, sensitivity and interpretability of model-based approaches for automated flow cytometry analysis. We demonstrate the consistency of frequency of HDPGMM estimates on reference data samples spiked with extremely low frequencies of antigen-specific cells, a scenario directly relevant to many clinical research studies in vaccine development, immune monitoring and immune biomarker discovery where the frequency of rare antigen-specific T cells is of interest.

## Methods

### Hierarchical modeling

#### Dirichlet process mixture of Gaussians

Assume we observe flow cytometry measurements 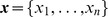 where each 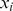 is a 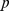 dimensional vector. Let the probability density function for 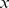 be
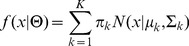
(1)where 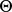 is the complete set of parameters in the model, 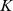 is the number of possible clusters, and 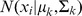 is the 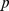 dimensional multivariate normal density evaluated at 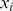 with mean 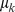 and covariance matrix 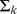. The mixture weights 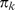, are all positive and sum to one. The 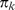 are modeled as random probabilities from a so-called “stick-breaking” prior process. Specifically,
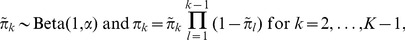
(2)where Beta denotes a beta distribution [17]. Note that 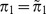 and 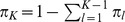. A key advantage of the (truncated) Dirichlet process specification is that it results in automated inference on the number of clusters based on a pre-specified large value 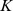. That is, with such an encompassing 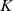, many of the 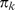 will be inferred as very close to zero, leaving a reduced set of effective clusters. A complete Bayesian model specification is completed by putting priors on 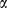, 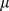, and 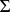.

An alternative and equivalent representation of (1) is to assume that for each observation 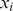 we have an unknown label 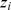. If we assume 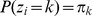 and 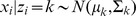, marginalizing the 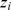 yields (1). This parametrization makes posterior computation more tractable, and inference about 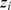 is equivalent to inferring the cluster assignment for 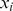.

#### Hierarchical Dirichlet process mixture of Gaussians

We now generalize DPGMM to simultaneously classify T cells across multiple datasets. Assume we observe 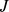 different sets of FCM measurements 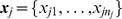. Each dataset then has its own probability density function given by
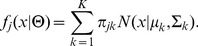
(3)Note the primary difference between DPGMM and HDPGMM is 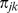. The sets of cluster locations and shapes, 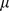 and 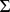, are assumed to be the same across datasets while the prevalence of the clusters 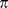 is allowed to vary across datasets. A similar two level parametrization holds here as in DPGMM. The approach now introduces the inherent, latent component indicators 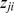 such that, for each observation 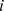 and component 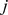, 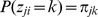; this leads to conditional distributions 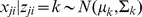 and opens the path to routine computational methods.

Our interest is in extensions of this basic framework to hierarchical models on the 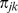 that effectively picks the number of clusters in the model, but shares information across datasets to facilitate rare subtype discovery. Teh et al (2006) [15] give multiple representations for a hierarchical Dirichlet process for clustering across multiple datasets. We take their stick breaking approach:
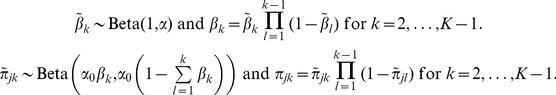
(4)As before, 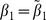, 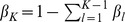, 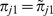, and 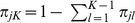. Teh et al (2006) [15] show that this construction is equivalent to letting each dataset have its own Dirichlet process where the base measures also arise from a common base measure. We complete the prior specification by placing multivariate normal and inverse Wishart priors on each 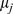 and 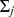 respectively. We also use gamma priors on 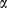 and 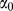 [11].

In summary, within each sample every cell is assumed to come from some unknown cluster where the number of clusters is learned from the data and the shape of each cluster is unknown. Note that we can assume this to be true because we group many parametric Gaussian clusters into flexibly shaped groups. See the consensus modal clustering below. Since the model is hierarchical in the sense that cluster shapes are shared between samples while their prevalence variance between samples, information is shared when cells from multiple samples are assigned to the same cluster giving us more information about the cluster's shape. This is especially prevalent when the number of cells in a particular cluster is small for a given sample.

#### Posterior computation

We perform posterior inference by sampling via a Markov chain Monte Carlo (MCMC) algorithm using the latent classification variable 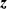. However, several challenges arise. First, the usual Gibbs sampling approach does not work because the conditional distributions of the 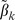 are difficult to work with. Crepet et al (2011) [18] use a similar model, but do not give details for sampling these key parameters. Secondly, the naming, or labeling, of the clusters is not well defined, so we need to deal with relabeling issues. Finally, the computation within each sampling iteration is very expensive.

#### Metropolis within Gibbs

Since the conditional distributions for 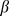 and 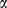 are not conjugate, we propose a Metropolis within Gibbs approach. For each MCMC iteration, all other parameters are sampled via their full conditional distributions given in the HDPGMM implementation section below. We then propose a new 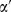 from a normal distribution centered at 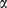 where we reflect negative values onto the positive half line and accept or reject the move according to the Metropolis Hastings (MH) ratio. We take the same approach for each 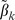 except that we reflect onto the unit interval. The variability in the random walks is tuned during the burn-in period to target a 50% acceptance rate supported by Gelman et al [19].

#### Identification

To address the label switching issue, we use the method of Cron and West [20] that maintains a coherent classification of the data across the MCMC iterates. This is enabled by defining a “reference” classification taken from the last iteration of the burn-in phase of the MCMC; this is simply the most likely cluster assignment for each event in all the datasets at that iterate. This labeling is chosen as a reference since it is assumed to be a representative labeling of the cells. Then, at every further iteration the clusters are relabeled to minimize the misclassification rate when compared to the reference. In essence, we pick a representative clustering then we choose the cluster labelings at every iterate that labels the data most like the reference. This method is used because of its computational efficiency and good performance in other settings. Critically, this allows us to estimate the true posterior by component-wise averaging over multiple iterations after the burn-in phase, instead of using a point estimate as is typically done. Complete details are given by Cron and West [20] including a flow cytometry example.

#### GPU computation

In each iteration of the MCMC, the multivariate normal distribution must be evaluated at every event (in every dataset) for each of the 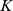 clusters to get assignment probabilities. Without parallel computing, this takes the majority of the computation time. Therefore, we adapted the GPU computing ideas by Suchard et al [16], [21] used in the “gpustats” python library to accelerate the computation. We also employed MPI techniques that use multiple GPUs across potentially multiple machines simultaneously to optimize performance.

#### Consensus modal clustering for cell subset identification and alignment

As cell subsets may have non-Gaussian distributions, it is often necessary to merge several mixture components to represent a single cell subtype. An intuitively appealing concept is to cluster components together when the components share a common mode, since the mode is an objective feature of the posterior distribution that links multiple components - here we adapt the procedure to find a coherent modal assignment across data sets. We first create a reference distribution whose whose components have the same means and covariance matrices as the fitted HDPGMM model, but whose component weights are averaged over all data sets. We first create a consensus Gaussian mixture model distribution whose components have the same means and covariance matrices as the fitted HDPGMM model, but whose component weights are averaged over all data sets. Starting from the location of each component mean, we use a numerically efficient iterative procedure to identify the mode associated with that location as previously described [10]. Components in the consensus GMM that approach the same mode to within a small tolerance are then merged to create a mapping of Gaussian components to modal clusters. The mapping is then used for all the fitted data sets, resulting in cell subset (modal cluster) alignment across multiple data sets. Note that only the *mapping* of component to modal cluster is shared by all data sets, the component weights for each data set remain unique.

### HDPGMM implementation

We give posterior computational details only for HDPGMM since details for our implementation of DPGMM have been previously published [16]. First, let 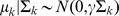 and 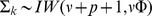 so that 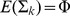. Furthermore, let 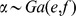 and 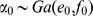. These along with equations (3) and (4) give a complete specification of the model. Metropolis within Gibbs is performed by updating each parameter with a draw from its conditional distribution in turn and when the conditional distribution is intractable, use a Metropolise Hastings update instead. We give the specifics of the sampling in the remainder of this section.

#### Sampling classification indicators

For each observation calculate
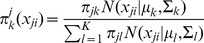
then sample each 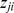 with pmf
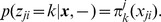
Note that we use the shorthand “
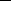
” to denote all other parameters in the model. This calculation occupies most of the computational effort for large datasets. However, this is completely parallelizable across observations and datasets. To achieve very large performance gains, we place one or more datasets 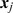 on each GPU available and use “gpustats” to perform the computations similar to [16].

#### Sampling cluster parameters

Given the cluster assignments, sampling each 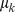 and 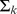 is simply a matter of drawing from their conjugate normal and inverse Wishart distributions respectively. Let, 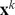 be all observations such that 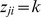 and 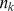 be the number of said observations. Sample
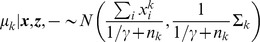
and
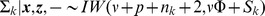
where 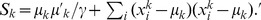


Updating the cluster weights, 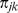, is slightly less routine but still conjugate. Define,
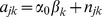


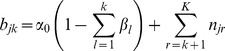
where 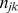 is the number of points in dataset 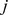 assigned to cluster 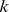. Sample 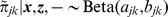.

Furthermore, 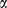 can be updated simply by
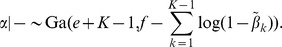



#### Metropolis Hasting sampling

The conditional distributions for 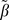 and 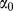 are not tractable for sampling directly. Therefore, we use Metropolis Hastings samples with reflected normal proposals for each 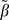 and 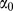 in turn. The algorithm is essentially the same in both cases, so we give a general description of the approach for sampling the parameter of interest, 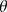, constrained between 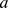 and 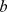. Note that 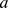 or 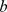 can be 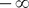 or 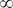 respectively.

First, sample 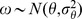 and set 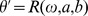 where 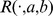 recursively reflects 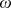 over the bounaries. While the pdf for this proposal distribution 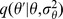 is tedious to write analytically, it can be shown that 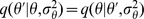. Finally, set 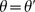 with probability
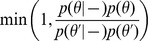
and leave 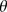 unchanged otherwise. Note that the proposal distribution does not enter the probability because it is symmetric. To choose the appropriate 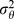, we track the acceptance rate during the burn in period. On a fixed interval, we check if the acceptance rate is above 50% or below 40% and reduce or increase 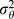 respectively. When sampling 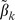,
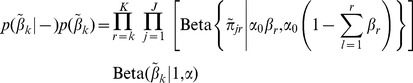
and 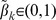. For 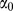,
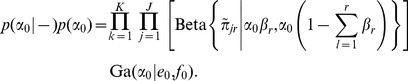



### Generation of experimental data and data preprocessing

The generation of the standard samples with a defined number of antigen-specific CD8 T cells spiked into autologous PBMC for use in HLA-peptide multimer has been described [8]. Briefly, Phytohemagglutinin (PHA; 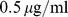) and IL-2 (20 U/ml) stimulated HLA-A*0201 positive PBMC were retrovirally transduced with an HLA-A*0201 restricted 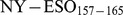 specific TCR construct after the CD4 T cells were depleted using Dynabeads (Invitrogen). After 5 days, the transduced cells were harvested and purified using APC-conjugated NY-ESO-1 specific HLA multimer and magnetic cell sorting. Purified cells were clonally expanded, harvested and spiked at the desired percentage of NY-ESO-1 specific TCR expressing CD8 T cells into autologous PBMC. These samples were stained with monoclonal antibodies specific for CD45 (pan leukocyte) CD3 (T-lymphocytes) and HLA-A*0201 NY-ESO-1 157–165 multimers to identify spiked T cells. For details, please refer to reference [8]).

Sample preparation conditions were set so that results (i.e. generated FCS files) would be as comparable as possible: Cell staining was performed simultaneously by the same operator, using the same batches of staining reagents, and data acquisition was subsequently done in a single experiment using the same cytometer settings (voltages, compensations) for all samples. The data were generated using a FACSCalibur and CellQuest Pro 6.0, with values ranging from 0 to 1023. No further transformations were performed on the data but standardization to have zero mean and unit standard deviation was performed before fitting the mixture model so all markers would have equal contributions. The standardization was reversed before plotting - i.e. all plots are based on the original 0 to 1023 scale. For gating estimates, frequency estimates from 10 flow cytometry operators using the same gating strategy were collected.

## Supporting Information

Figure S1 **Comparison of HDPGMM, FLAME and flowClust with same number of mixture components.** The panels show the estimated frequencies of antigen-specific cells (large red dots) expressed as a percentage of all events (yellow boxes). (Left panel) HDPGMM detects the antigen-specific cluster at all spiked-in frequencies with cell subset alignment as indicated by the color coding of events. (Middle panel), FLAME identified antigen-specific cell subsets at spiked-in frequencies of 0.02625% of greater, but the alignment stage failed with an error message and hence clusters are not aligned. (Right panel) FlowClust failed to identify any antigen-specific clusters and cell subsets are not aligned. Note that both HDPGMM and FLAME detect a moderate number of CD3-negative false positive events, suggesting that 48 components are insufficient to adequately model rare event subsets in this data set.
(TIF)

Text S1 Text S1 contains instructions on how to install the software and run the examples provided.
(ZIP)

Software S1 Software S1 contains a Makefile, source code and scripts to generate the figures shown in the manuscript.
(ZIP)

## References

[1] Britten C, Gouttefangeas C, Welters M, Pawelec G, Koch S, et al (2008) The CIMT-monitoring panel: a two-step approach to harmonize the enumeration of antigen-specific CD8+ T lymphocytes by structural and functional assays. Cancer Immunology, Immunotherapy 57: 289–302. 17721783 10.1007/s00262-007-0378-0 PMC2150627

[2] Welters MJP, Gouttefangeas C, Ramwadhdoebe TH, Letsch A, Ottensmeier CH, et al (2012) Harmonization of the intracellular cytokine staining assay. Cancer Immunology, Immunotherapy 61: 1–12. 22714399 10.1007/s00262-012-1282-9 PMC3378841

[3] Aghaeepour N, Finak G, Hoos H, Mosmann TR, Brinkman R, et al (2013) Critical assessment of automated ow cytometry data analysis techniques. Nature methods 10: 228–238. 23396282 10.1038/nmeth.2365 PMC3906045

[4] Chan C, Feng F, Ottinger J, Foster D, West M, et al (2008) Statistical mixture modeling for cell subtype identification in ow cytometry. Cytometry Part A 73A: 693–701. 10.1002/cyto.a.20583 PMC2840702 18496851

[5] Pyne S, Hu X, Wang K, Rossin E, Lin T, et al (2009) Automated high-dimensional ow cytometric data analysis. Proceedings of the National Academy of Sciences 106: 8519. 10.1073/pnas.0903028106 PMC2682540 19443687

[6] Lo K, Hahne F, Brinkman R, Gottardo R (2009) flowClust: a Bioconductor package for automated gating of ow cytometry data. BMC Bioinformatics 10: 145. 19442304 10.1186/1471-2105-10-145 PMC2701419

[7] Scheuermann R, Qian Y, Wei C, Sanz I (2009) ImmPort FLOCK: Automated cell population identification in high dimensional ow cytometry data. The Journal of Immunology 182: 42–17.

[8] Singh S, Tummers B, Schumacher T, Gomez R, Franken K, et al (2012) The development of standard samples with a defined number of antigen-specific T cells to harmonize T cell assays: a proof-of-principle study. Cancer Immunology, Immunotherapy 62: 1–13. 22986454 10.1007/s00262-012-1351-0 PMC3589624

[9] Finak G, Bashashati A, Brinkman R, Gottardo R (2009) Merging mixture components for cell population identification in flow cytometry. Advances in Bioinformatics 2009: Article ID 247646. 10.1155/2009/247646 PMC2798116 20049161

[10] Chan C, Lin L, Frelinger J, Hebert V, Gagnon D, et al (2010) Optimization of a highly standardized carboxyuorescein succinimidyl ester ow cytometry panel and gating strategy design using discriminative information measure evaluation. Cytometry Part A 77: 1126–1136. 10.1002/cyto.a.20987 PMC3042236 21053294

[11] Escobar MD, West M (1995) Bayesian density estimation and inference using mixtures. Journal of the American Statistical Association 90: 577–588.

[12] Lin L, Chan C, Hadrup SR, Froesig TM, Wang Q, et al (2013) Hierarchical bayesian mixture modelling for antigen-specific T-cell subtyping in combinatorially encoded ow cytometry studies. Statistical Applications in Genetics and Molecular Biology 1–23. 23629459 10.1515/sagmb-2012-0001 PMC4155753

[13] Müller P, Quintana F, Rosner G (2004) A method for combining inference across related nonparametric Bayesian models. Journal of the Royal Statistical Society: Series B (Statistical Methodology) 66: 735–749.

[14] de Oliveira Sales AP (2011) Clustering Multiple Related Datasets with a Hierarchical Dirichlet Process. Master's thesis, Duke University.

[15] Teh YW, Jordan MI, Beal MJ, Blei DM (2006) Hierarchical Dirichlet processes. Journal of the American Statistical Association 101: 1566–1581.

[16] Suchard MA, Wang Q, Chan C, Frelinger J, Cron AJ, et al (2010) Understanding GPU programming for statistical computation: Studies in massively parallel massive mixtures. Journal of Computational and Graphical Statistics 19: 419–438. 20877443 10.1198/jcgs.2010.10016 PMC2945379

[17] Ishwaran H, James L (2001) Gibbs sampling methods for stick-breaking priors. Journal of the American Statistical Association 96: 161–173.

[18] Crepet A, Tressou J (2011) Bayesian nonparametric model for clustering individual co-exposure to pesticides found in the French diet. Bayesian Analysis 6: 127–144.

[19] Gelman A, Roberts GO, Gilks WR (1996) Efficient Metropolis jumping rules. Bayesian Statistics 5: 599–607.

[20] Cron AJ, West M (2011) Efficient classification-based relabeling in mixture models. The American Statistician 65: 16–20. 21660126 10.1198/tast.2011.10170 PMC3110018

[21] Suchard MA, Holmes C, West M (2010) Some of the What?, Why?, How?, Who? and Where? Of graphics processing unit computing for Bayesian analysis. Bulletin of the International Society for Bayesian Analysis 17: 12–16.

