# Supplementary figures and images for: Hierarchical Modeling for Rare Event Detection and Cell Subset Alignment across Flow Cytometry Samples
Source: PLoS Comput Biol. 2013 Jul 11;9(7):e1003130. doi: 10.1371/journal.pcbi.1003130 (PMC3708855; doi:10.1371/journal.pcbi.1003130)

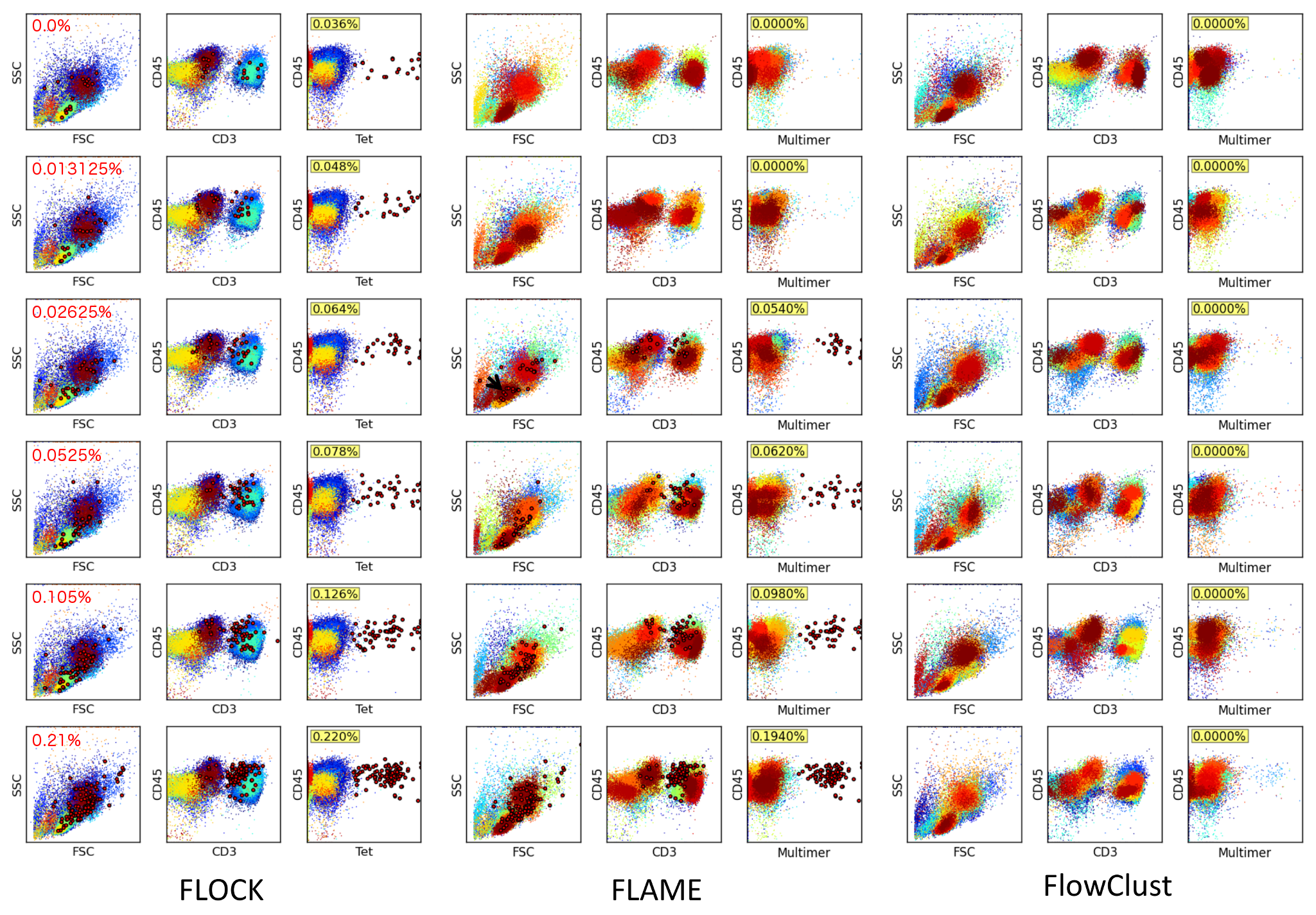

Supplement: Figure S1 — Comparison of HDPGMM, FLAME and flowClust with same number of mixture components. The panels show the estimated frequencies of antigen-specific cells (large red dots) expressed as a percentage of all events (yellow boxes). (Left panel) HDPGMM detects the antigen-specific cluster at all spiked-in frequencies with cell subset alignment as indicated by the color coding of events. (Middle panel), FLAME identified antigen-specific cell subsets at spiked-in frequencies of 0.02625% of greater, but the alignment stage failed with an error message and hence clusters are not aligned. (Right panel) FlowClust failed to identify any antigen-specific clusters and cell subsets are not aligned. Note that both HDPGMM and FLAME detect a moderate number of CD3-negative false positive events, suggesting that 48 components are insufficient to adequately model rare event subsets in this data set. (TIF) [file pcbi.1003130.s001.tif]
